# Genome-wide run of homozygosity analysis reveals candidate genomic regions associated with environmental adaptations of Tibetan native chickens

**DOI:** 10.1186/s12864-021-08280-z

**Published:** 2022-01-31

**Authors:** Jingwei Yuan, Shijun Li, Zheya Sheng, Meikun Zhang, Xuming Liu, Zhengdong Yuan, Ning Yang, Jilan Chen

**Affiliations:** 1 Institute of Animal Sciences, Chinese Academy of Agricultural Sciences, Beijing, 100193 China; 2 Key Laboratory of Agricultural Animal Genetics, Breeding and Reproduction of Ministry of Education, Huazhong Agricultural University, Wuhan, 430070 China; 3 DQY Ecological Co. Ltd., Beijing, 100094 China; 4 National Engineering Laboratory for Animal Breeding, College of Animal Science and Technology, China Agricultural University, Beijing, 100193 China

**Keywords:** Tibetan native chicken, Genetic diversity, Run of homozygosity, Genomic inbreeding coefficient, Candidate genes, Adaptation

## Abstract

**Background:**

In Tibet, the two most important breeds are Tibetan chicken and Lhasa white chicken, and the duo exhibit specific adaptations to the high altitude thereby supplying proteins for humans living in the plateau. These breeds are partly included in the conservation plans because they represent important chicken genetic resources. However, the genetic diversity of these chickens is rarely investigated. Based on whole-genome sequencing data of 113 chickens from 4 populations of Tibetan chicken including Shigatse (SH), Nyemo (NM), Dagze (DZ) and Nyingchi (LZ), as well as Lhasa white (LW) chicken breed, we investigated the genetic diversity of these chicken breeds by genetic differentiation, run of homozygosity (ROH), genomic inbreeding and selection signature analyses.

**Results:**

Our results revealed high genetic diversity across the five chicken populations. The linkage disequilibrium decay was highest in LZ, while subtle genetic differentiation was found between LZ and other populations (Fst ranging from 0.05 to 0.10). Furthermore, the highest ROH-based inbreeding estimate (F_ROH_) of 0.11 was observed in LZ. In other populations, the F_ROH_ ranged from 0.04 to 0.06. In total, 74, 111, 62, 42 and 54 ROH islands containing SNPs ranked top 1% for concurrency were identified in SH, NM, DZ, LZ and LW, respectively. Genes common to the ROH islands in the five populations included *BDNF*, *CCDC34*, *LGR4*, *LIN7C*, *GLS*, *LOC101747789*, *MYO1B*, *STAT1* and *STAT4*. This suggested their essential roles in adaptation of the chickens. We also identified a common candidate genomic region harboring *AMY2A*, *NTNG1* and *VAV3* genes in all populations. These genes had been implicated in digestion, neurite growth and high-altitude adaptation.

**Conclusions:**

High genetic diversity is observed in Tibetan native chickens. Inbreeding is more intense in the Nyingchi population which is also genetically distant from other chicken populations. Candidate genes in ROH islands are likely to be the drivers of adaptation to high altitude exhibited by the five Tibetan native chicken populations. Our findings contribute to the understanding of genetic diversity offer valuable insights for the genetic mechanism of adaptation, and provide veritable tools that can help in the design and implementation of breeding and conservation strategies for Tibetan native chickens.

**Supplementary Information:**

The online version contains supplementary material available at 10.1186/s12864-021-08280-z.

## Background

The Tibetan plateau is the largest high-altitude area on earth with an average altitude exceeding 4000 m, representing 25% of the landmass of China. This high-altitude environment becomes a habitat for many unique animal genetic resources. Tibetan chicken is one of the chicken breeds found in this area dating back to the seventh century A.C [1]. The chickens are widely distributed in farming areas of Tibet, including Shigatse, Lhasa, Lhoka and Nyingchi. Beside Tibetan chickens, Lhasa white chicken is another widespread native breed reared on the plateau, which is bred for several decades by intercrossing male White Leghorn and female Tibetan chickens. The basis of physiological and genetic adaptations to the extreme environmental conditions of the Tibetan plateau have recently been partly investigated in Tibetan chickens [1–3], and the genomic analysis indicated that the chickens might be a composite of multiple distinct populations [2, 4]. However, population genomic analysis was rarely conducted to explore the diversity of the chicken populations reared in Tibet and to guide genetic resource conservation and utilization efforts.

The availability of high-throughput affordable sequencing techniques has allowed genome-wide analysis of the genetic structure and relationships in animal populations. Large scale omics data have opened new perspectives for a more accurate animal genetic analysis. In addition to SNP and gene expression data, runs of homozygosity (ROH) has joined the assembly of omics data available in biological databases for livestock gene discovery and diversity assessment. ROH is a kind of long continuous homozygous stretches in the genome that are formed by the combination of two identical haplotypes in an individual [5]. These regions are ubiquitous even in outbred populations, and are usually considered to be the index of autozygosity. Long homozygous regions throughout the genome result from demographic events, mating between close relatives (population bottleneck), reduction in population size (leading to be more likely exposed to genetic drift), selection (breeding) and small inversions that suppress recombination events. Thus, population demography, structure and diversity can be explored based on the distribution and location of ROH regions of the genome. Moreover, previous studies have shown that ROH-based inbreeding estimates provide a better measure of individual autozygosity than pedigree-based estimates of overall inbreeding if kinship between founder animals are not accounted [6]. The occurrence of ROH in individual genomes has also facilitated our understanding of the genetic basis of complex phenotypes. ROH analyses enabled the study of genomic regions with high incidence of homozygosity across individuals, which were first referred to as ROH islands [7] within a population. In addition, the ROH islands can provide important insights into the population signatures of positive selection due to the linkage disequilibrium (LD) [8]. Hence, overlapping ROH islands across populations and species are valuable in comparative genomic studies and may reveal critical genetic regions for complex traits. Consequently, ROH analyses are becoming complementary to genome-wide association studies in the detecting population-specific major genes in humans and animals [9].

In chickens, analysis of ROH has been deployed to assess the genome-wide diversity in local and imported genetic resources. Results from such analysis help in the design and review of effective conservation program for endangered populations [10–13]. The long-term selection molded the presence of ROHs and their associated genomic regions resulting in unique population adaptation to environment-imposed challenges in broilers, suggesting that ROHs might also be a product of selection events rather than demographic and population history [14]. Moreover, multiple candidate genes involved in growth, egg production, disease resistance and behavior were associated with the ROH islands in different chicken breeds [15, 16].

Within-breed diversity and the population structure analyses in livestock species are fundamental for understanding environmental adaptation, implementing conservation programs and designing selection plans [10, 17, 18]. While a huge effort was expended to study cosmopolitan breeds in the past, a growing attention had been shifted to the local breeds which are important genetic resources for their potential to solving problems in agriculture related to environmental changes [19, 20]. Local chickens in Tibet have evolved over centuries under extreme natural conditions. They may serve as a great reservoir of the genetic pool for the identification of genes under natural and artificial selection particularly those harboring putative signatures of environmental adaptation. Herein, four Tibetan chicken populations and a local cultivated breed (Lhasa white) were sampled in this study during 2016 and 2017. All the Tibetan chicken populations were raised traditionally by local farmers, while Lhasa white was managed at the Institute of Animal Husbandry and Veterinary, Tibetan Academy of Agricultural and Animal Husbandry Sciences. The objectives of the present study were to (i) evaluate the genetic diversity of Tibetan chickens reared in different areas of the Tibetan plateau using whole-genome sequencing data, (ii) detect ROH within each chicken population and evaluate the genomic inbreeding and (iii) reveal the genomic regions of ROH islands that may influence the adaptation of Tibetan native chicken to high altitude. The results are expected to provide valuable information for the gene-phenotype association, as well as for the conservation of chicken genetic resources in Tibet.

## Results

### Summary of the genetic diversity parameters

The genetic diversity for Shigatse (SH), Neymo (NM), Dagze (DZ), Nyingchi (LZ) and Lhasa white (LW) chicken populations was evaluated by observed heterozygosity (Ho), expected heterozygosity (He) and multiple-locus heterozygosity (MLH) using eligible SNPs under Hardy–Weinberg equilibrium. Ignoring the minor allele frequency (MAF) of SNPs, the Ho ranged from 0.15 to 0.18. Using filtered SNPs with MAF ≥ 5%, Ho increased ranging from 0.27 to 0.31. The estimates of He were similar to Ho for each population. MLH ranged from 0.22 to 0.31 when all SNPs were used, and it went up from 0.27 to 0.31 when only SNPs with MAF ≥ 5% were used. The MAF ranged from 0.11 to 0.13 when its filter was set at 0, and it went up from 0.21 to 0.22 when only SNPs with MAF ≥ 5% were used. The diversity indices of the five populations were shown in Table 1. The linkage disequilibrium (LD) decay pattern was different in the five populations (Fig. S1a). Strong LD was observed between SNPs within a short range in all five populations. For SNPs up to 5 kb apart, the average r^2^ values were 0.08, 0.07, 0.08, 0.20 and 0.09 for SH, NM, DZ, LZ and LW populations, respectively. This indicated a stronger LD between SNPs and a clear difference in LZ compared to the other four chicken populations. Furthermore, the analysis of population relatedness (Table S1) revealed that Fst values were small for all pairwise comparisons. The lowest value of 0.001 was observed in SH vs. LW while the highest value of 0.095 was observed in DZ vs. LZ. Concordantly, the LZ population was moderately distant from other populations with a larger Fst value ranging from 0.052 to 0.095, while the LW population was a synthesized line which seemed to be genetically closest to all Tibetan chicken populations. All eligible SNPs filtered for HWE and call rate were used for principal component analysis (PCA), which revealed a cluster separation between LW and the other 4 Tibetan chicken population. SH, LZ and DZ were also clearly separated by PC1 and PC2, except for a few scattered individuls. Chickens from NM population mixed together with DZ, occupying an intermediate position between SH and DZ populations (Fig. S1b).

**Table 1:** Observed (Ho), expected (He) heterozygosity and minor allele frequency (MAF) for each chicken population

Population	N		ALL SNPs				SNPs with MAF ≥ 0.01				SNPs with MAF ≥ 0.05		
		Ho	He	MLH	MAF	Ho	He	MLH	MAF	Ho	He	MLH	MAF
SH	20	0.17 ± 0.17	0.19 ± 0.17	0.23 ± 0.02	0.13 ± 0.14	0.23 ± 0.17	0.24 ± 0.16	0.23 ± 0.02	0.17 ± 0.14	0.27 ± 0.15	0.29 ± 0.14	0.27 ± 0.03	0.21 ± 0.14
NM	32	0.18 ± 0.17	0.18 ± 0.17	0.22 ± 0.02	0.13 ± 0.14	0.23 ± 0.16	0.22 ± 0.16	0.23 ± 0.02	0.16 ± 0.14	0.30 ± 0.14	0.31 ± 0.13	0.30 ± 0.02	0.22 ± 0.13
DZ	25	0.18 ± 0.18	0.18 ± 0.17	0.23 ± 0.04	0.13 ± 0.14	0.23 ± 0.17	0.24 ± 0.16	0.23 ± 0.04	0.17 ± 0.14	0.31 ± 0.15	0.31 ± 0.13	0.31 ± 0.05	0.22 ± 0.13
LZ	10	0.15 ± 0.20	0.15 ± 0.18	0.31 ± 0.01	0.11 ± 0.15	0.31 ± 0.18	0.30 ± 0.15	0.31 ± 0.01	0.22 ± 0.14	0.31 ± 0.18	0.30 ± 0.15	0.31 ± 0.01	0.22 ± 0.14
LW	18	0.18 ± 0.18	0.18 ± 0.18	0.25 ± 0.05	0.13 ± 0.14	0.25 ± 0.17	0.25 ± 0.16	0.25 ± 0.05	0.18 ± 0.14	0.29 ± 0.16	0.30 ± 0.14	0.29 ± 0.05	0.21 ± 0.13

Note: SH, NM, DZ, LZ and LW denote Shigatse, Nyemo, Dagze, Ningychi and Lhasa white chicken population, respectively. Ho, He, MLH and MAF denote observed heterozygosity, expected heterozygosity, multiple-locus heterozygosity and minor allele frequency, respectively. Data was shown as mean ± standard deviation

### Runs of homozygosity within the population

**Fig. 1:**
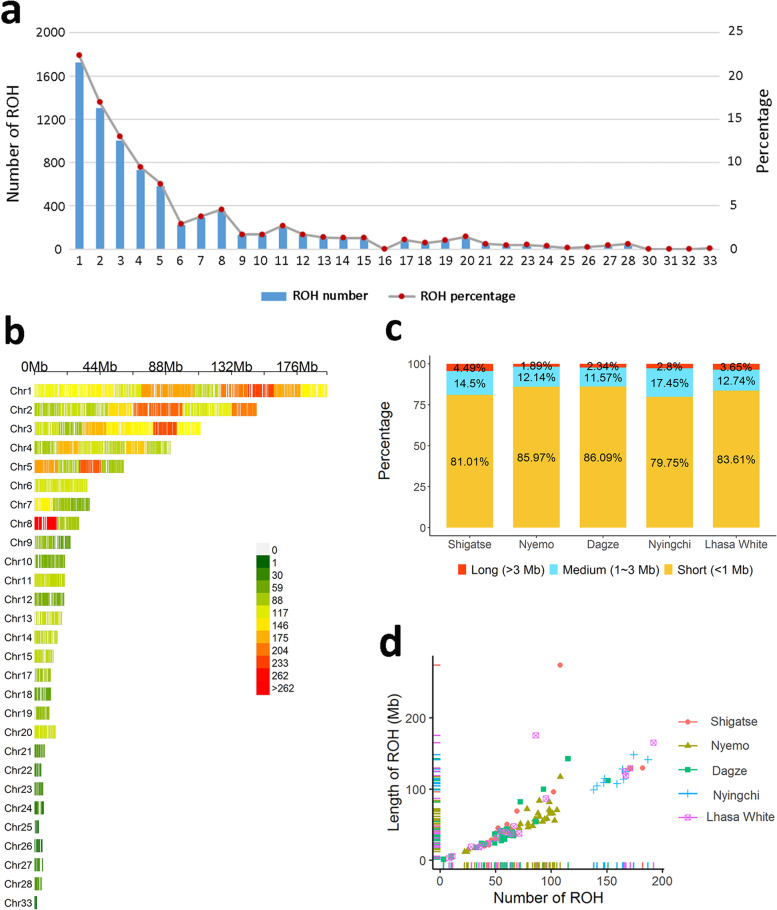
Descriptive graphics of run of homozygosity (ROH) in 5 Tibetan native chicken populations. **a** The average number of ROHs per chromosome (bars) and the average percentage of each chromosome covered by ROHs (lines) for all chickens. **b** The distribution and density of ROHs in the whole genome for all chickens. The different color represents different number of ROHs within 18 Mb window size. **c** The percentage of total ROH within each ROH length category, including short (< 1 Mb), medium (1–3 Mb), and long (> 3 Mb) per chicken population. **d** Total number of ROHs and total length of genome (Mb) covered by ROH segments per birds for each chicken population

The current study identified 1269, 2438, 1366, 1284 and 1342 ROHs in SH, NM, DZ, LZ and LW chicken population, respectively. ROHs were identified in each sampled bird. Population-wise, the average number of ROH segments was highest in the LZ (128.4 ROH/bird) compared to the other populations. The lowest and highest average length of ROHs were observed in DZ (54.64 Mb) and LZ (102.54 Mb), respectively. The number of SNPs harbored in the ROHs varied between the studied populations, and the maximum number (22,386) was located on GGA1 of the SH population. Similarly, the minimum number of SNPs in the ROHs (50) was located on GGA1 found in the LZ chicken population (Table 2). As shown in Fig. 1a and b, ROHs identified in 106 birds were mainly distributed across the GGA1 to GGA15, GGA17 to GGA28 and GGA33 of the chicken genome. However, the majority of ROH regions were clustered in the macrochromosomes (GGA1 ~ GGA9). The classification of ROHs based on size shown that the short ROHs (< 1 Mb) were predominant, accounting for 79.75 to 86.09% of ROHs across all populations (Fig. 1c). For a better view, we plotted the ROH number against their size for each bird (Fig. 1d). We observed a consistently high correlation in NM (r = 0.87), DZ (r = 0.92), LZ (r = 0.91) and LW (r = 0.84), while correlation considerably varied in SH (r = 0.71) chickens. Moreover, the bird with an extremely long ROH (277.112 Mb) was found in the SH population, while the bird with the shortest ROH (0.831 Mb) belonged to the DZ population.

**Table 2:** Descriptive statistics of run of homozygosity for five chicken populations

Population	N	Number of ROH	Average number per bird	Length of ROH (Mb)	Average length per bird (Mb)	SNP number range
SH	20	1269	63.45 ± 41.94	1116.19	55.81 ± 62.57	52 ~ 22,386
NM	32	2438	73.88 ± 25.87	1680.94	50.94 ± 23.61	52 ~ 18,809
DZ	25	1366	54.64 ± 32.71	974.25	38.97 ± 34.86	50 ~ 17,675
LZ	10	1284	128.40 ± 12.69	1025.36	102.54 ± 15.05	50 ~ 2024
LW	18	1342	74.56 ± 55.92	1089.51	60.53 ± 54.94	50 ~ 13,096

Note: SH, NM, DZ, LZ and LW denote Shigatse, Nyemo, Dagze, Ningychi and Lhasa white chicken population, respectively. Data were shown as mean ± standard deviation

### Genomic inbreeding coefficients

**Fig. 2:**
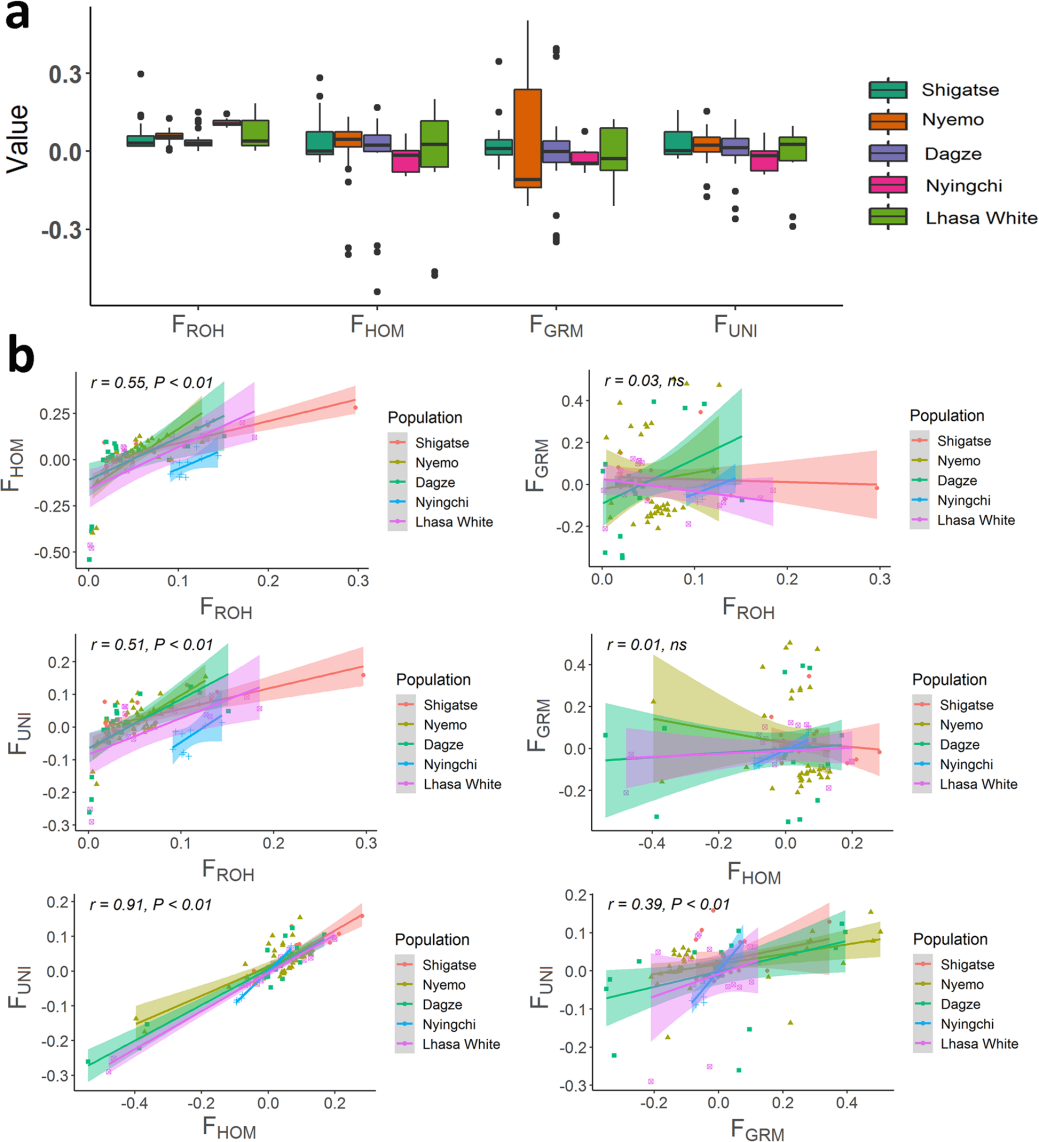
Genomic inbreeding and their correlation in 5 Tibetan native chicken populations. **a** Genomic inbreeding coefficients inferred from the proportion of the genome within ROH (F_ROH_), genomic SNP-by-SNP inbreeding coefficient (F_GRM_), excess of homozygosity (F_HOM_) and correlation between uniting gametes (F_UNI_). **b** The correlation between each of 2 genomic inbreeding coefficients across birds. The scatter plot was distinguished by chicken population

Genomic inbreeding was evaluated by the proportion of the genome within ROH (F_ROH_), genomic SNP-by-SNP inbreeding coefficient (F_GRM_), excess of homozygosity (F_HOM_) and correlation between uniting gametes (F_UNI_). As shown in Fig. 2a, the four genomic inbreeding coefficients were small and varied across the five populations. These coefficients were consistent within each population except for LZ, which showed high F_ROH_ (0.110 ± 0.016) and low F_HOM_ (− 0.029 ± 0.055), low F_GRM_ (− 0.027 ± 0.046) and low F_UNI_ (− 0.027 ± 0.052). We further analyzed the correlation among the four inbreeding coefficients which was 0.55 between F_ROH_ and F_HOM_, 0.51 between F_ROH_ and F_UNI_, 0.91 between F_HOM_ and F_UNI_, 0.39 between F_GRM_ and F_UNI_, respectively (Fig. 2b). However, the correlations between F_ROH_ and F_GRM_, and between F_HOM_ and F_GRM_ were not significant. Moreover, birds in the LZ population had consistent inbreeding coefficients for each index, whereas large inter-bird variability existed for each index in the LW population.

### Gene annotation of ROHs

**Fig. 3:**
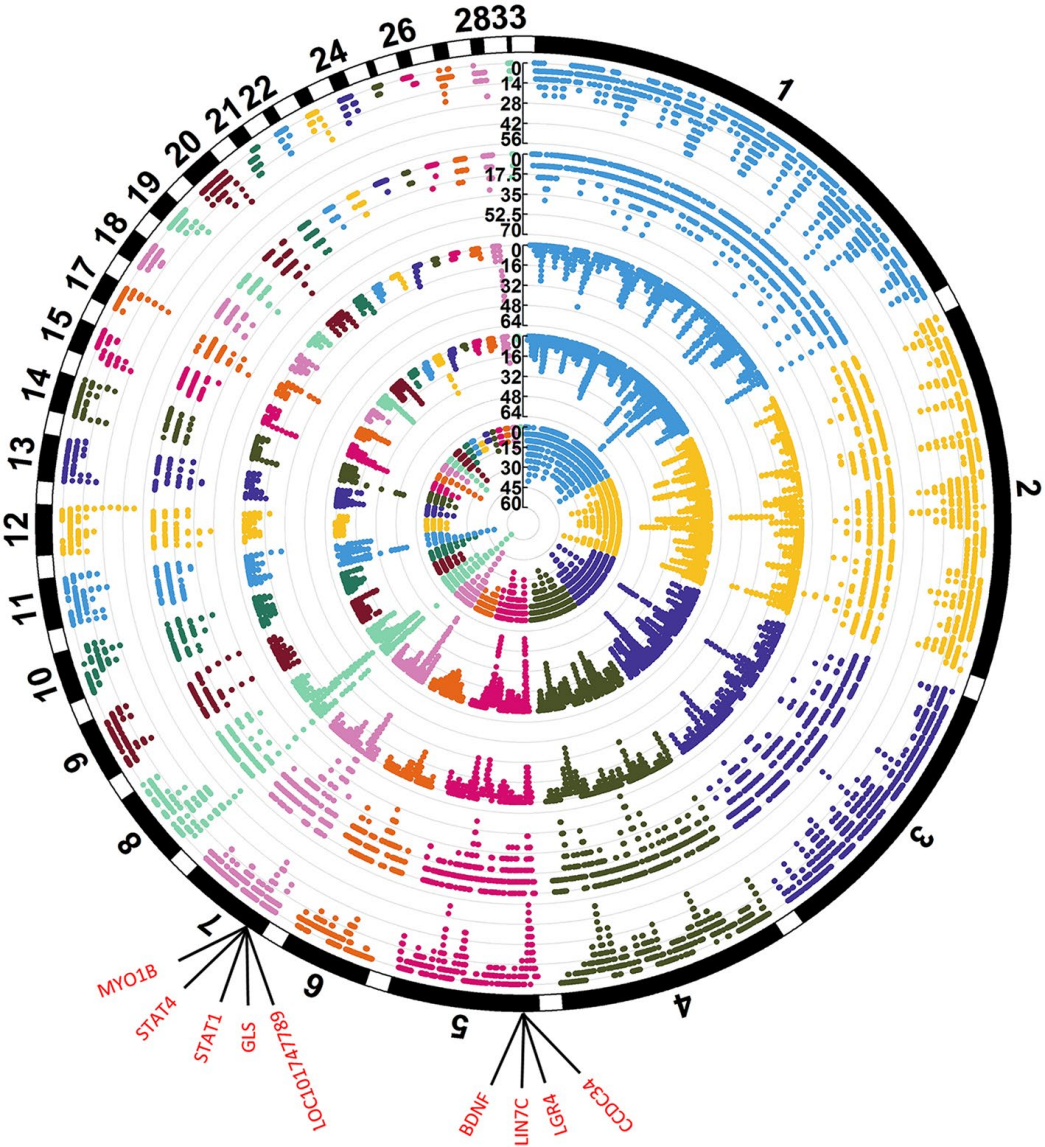
Circular Manhattan plot incidence of each SNP in run of homozygosity (ROH) for 5 Tibetan native chicken populations. From inside to outside, circles denote Shigatse, Nyemo, Dagze, Ningychi and Lhasa white population, respectively. The outermost circle denotes the chromosome. The shared genes harbored in the top 1% ROH islands by five populations were shown in red. The y axis denotes the frequency (%) of SNP that occurred in ROH

We detected 74, 111, 62, 42 and 54 ROH islands that ranked top 1% in SH, NM, DZ, LZ and LW chicken population, respectively (Fig. 3). Annotated genes within the ROH islands were retrieved from the Ensembl genome browser, resulting in 316, 491, 259, 197 and 166 genes in SH, NM, DZ, LZ and LW, respectively. Gene ontology (GO) analysis revealed that these genes were significantly enriched in the biological processes including positive regulation of synapse assembly, positive regulation of I-kappaB kinase/NF-kappaB signaling, osteoblast differentiation, cellular response to amino acid stimulus, cell adhesion and endodermal cell differentiation (Table S2). Among these genes, 11 were common to all the five populations and were located on GGA5 and GGA7. These genes include *BDNF*, *CCDC34*, *LGR4*, *LIN7C*, *GLS*, *LOC101747789*, *MYO1B*, *STAT1* and *STAT4* (Fig. 3). In addition, the top 1% ROH islands were mapped to the chicken QTL database and the ROH islands overlapped with 26, 29, 21, 19 and 17 QTLs in SH, NM, DZ, LZ and LW population, respectively (Table S3). Common QTLs that overlapped with the top 0.1% ROH islands in the five populations were for comb and ileum weight. Moreover, the ROH islands that harbored QTL for the ovary weight and percentage and wattle weight and length were specifically detected in the Tibetan chicken (Table 3).

**Table 3:** Top 0.1% ROH islands overlapped with reported QTLs for 5 populations

Population	Chromosome	Start (bp)	End (bp)	Number of SNPs	Associated QTL trait
SH	1	190,913,541	191,008,990	46	Feed intake
	2	144,110,859	144,201,790	45	Wattles weight
	3	37,006,451	37,110,698	54	Comb weight
	3	85,264,887	85,339,273	23	Comb weight
	3	85,362,178	85,530,321	89	Comb weight
	7	7,051,986	7,293,162	59	Ovary weight
	7	7,917,536	8,273,027	131	Ovary weight
	**8**	**31,868**	**702,746**	**267**	**Ileum weight**
	**8**	**742,239**	**797,216**	**54**	**Ileum weight**
	8	11,310,511	11,656,850	81	Ovary percentage
	11	2,781,379	3,782,297	125	Feed intake
NM	1	127,562,685	127,670,930	62	Bursa of Fabricius weight
	1	148,097,189	148,502,945	271	Fear-tonic immobility duration
	2	93,414,138	93,804,523	192	Feather-crested head
	3	37,019,870	37,444,374	202	Comb weight
	3	85,136,461	85,311,108	79	Comb weight
	3	85,335,481	85,477,712	77	Comb weight
	5	2,234,882	2,605,876	141	Body weight (28 days)
	7	8,089,149	8,139,910	12	Ovary weight
	7	7,239,376	8,061,927	104	Ovary weight
	**8**	**198,456**	**643,706**	**177**	**Ileum weight**
	11	3,367,829	3,766,928	130	Feed intake
	14	31,804	780,922	364	Wattles length
DZ	1	41,890,128	42,183,082	103	Yolk weight
	2	82,252,562	82,590,119	120	Feather-crested head
	2	82,995,651	83,236,647	99	Feather-crested head
	2	143,417,810	143,575,154	88	Wattles weight
	3	36,964,612	37,202,503	113	Comb weight
	3	37,203,796	37,293,935	33	Comb weight
	**8**	**37,270**	**401,505**	**108**	**Ileum weight**
	8	9,055,060	9,347,374	117	Ovary percentage
	8	9,712,707	10,369,442	222	Ovary percentage
	8	10,503,250	11,473,553	108	Ovary percentage
	8	11,475,230	11,998,950	129	Ovary percentage
	28	4,669,120	4,969,625	72	Abdominal fat weight
LZ	2	134,425,921	134,528,295	29	Wattles weight
	4	41,794,287	41,979,935	33	Ileum weight
	7	8,252,320	8,252,320	1	Ovary weight
	7	9,960,252	10,065,745	42	Ovary weight
	7	8,284,975	8,814,836	65	Ovary weight
	7	8,252,320	8,252,320	1	Comb weight
	7	9,960,252	10,065,745	42	Comb weight
	7	8,284,975	8,814,836	65	Comb weight, Receiving feather pecking
	7	9,960,252	10,065,745	42	Pectoralis minor weight
	**8**	**781,122**	**1,127,960**	**67**	**Ileum weight,** Body weight (36 days), Body weight (46 days), Spleen weight
	8	1,443,038	1,580,622	40	Ileum weight, Body weight (36 days), Body weight (46 days)
LW	1	127,516,561	127,828,483	102	Bursa of Fabricius weight
	1	150,304,925	150,605,645	120	Fear-tonic immobility duration
	1	160,765,702	160,956,991	87	Fear-tonic immobility duration
	2	73,319,835	73,368,670	11	Feather-crested head
	2	82,462,750	83,287,108	429	Feather-crested head
	3	60,530,655	60,739,692	107	Comb weight
	4	71,334,142	71,797,285	181	Gizzard weight
	**8**	**357,766**	**657,445**	**112**	**Ileum weight**
	12	19,551,518	19,936,175	171	Breast muscle pH

Note: SH, NM, DZ, LZ and LW denote Shigatse, Nyemo, Dagze, Ningychi and Lhasa white chicken population, respectively. ROHs highlighted in bold denote the shared QTL on chromosome 8 in 5 chicken populations

### Selection signature analysis

**Fig. 4:**
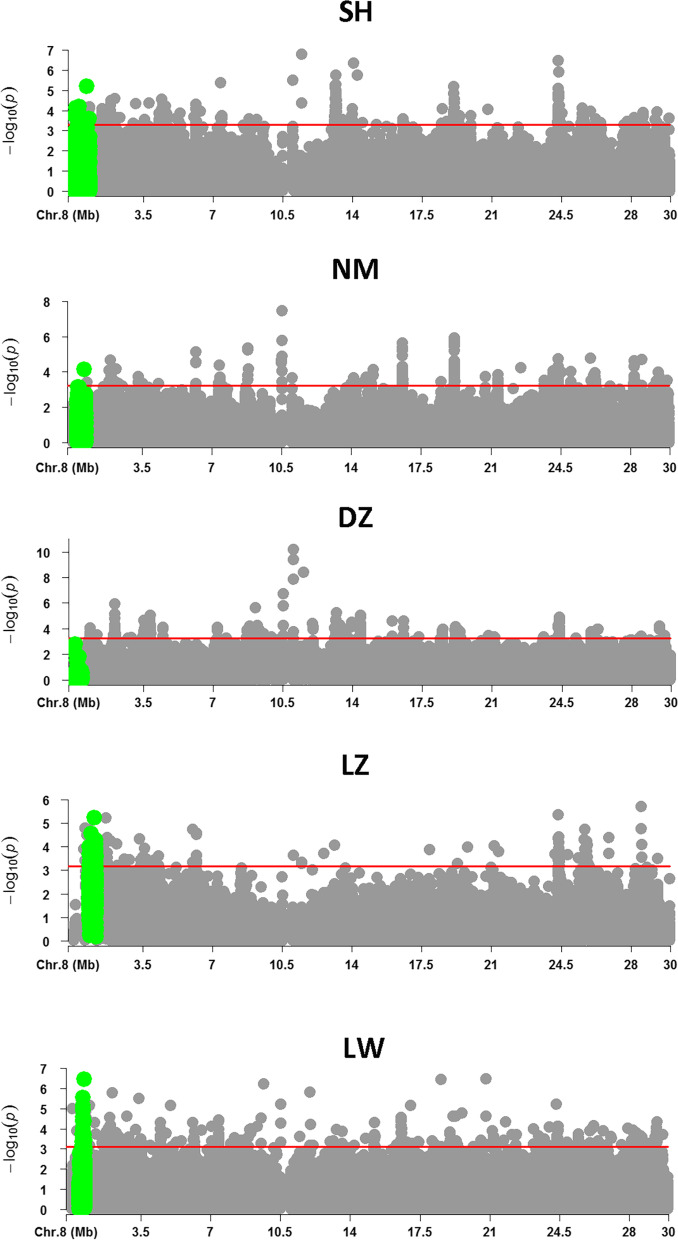
Chromosome-wide distribution of selection signatures detected by iHS on Chromosome 8 for 5 Tibetan native chicken populations. The red line represents the threshold levels of SNPs with iHS value ranked top 0.1%. The green dots represented SNPs located in the studied region (0.03 Mb to 1.13 Mb) and other dots located outside of the studied region were shown in grey. SH, NM, DZ, LZ and LW denote Shigatse, Nyemo, Dagze, Ningychi and Lhasa white chicken population, respectively

Notably, the genomic region containing common QTLs spanned from 0.03 Mb to 1.13 Mb of GGA8, and harbored six top 0.1% ROH islands across the five populations. By focusing on GGA8, we calculated integrated haplotype homozygosity (iHS) for each population. In all the populations except for the DZ population, SNPs harbored in the top 0.1% ROH islands were strongly selected for (Fig. 4), and the average |iHS| values of SNPs in each ROH island were higher than the average value of the total SNPs on GGA8. There were 1, 1, 8 and 2 SNPs (*P*-value ranked top 0.1%) that harbored signatures of selection in SH, NM, LZ and LW population, respectively. Further mining of this region revealed that only three genes (*AMY2A*, *NTNG1* and *VAV3*) were located within it. The information of ROH islands, SNPs and genes harbored within the studied region were listed in Table 4. In addition, we found similar results on GGA5, in which the share ROH island was also strongly selected (Fig. S2).

**Table 4:** The extended homozygosity of ROH island detected on chromosome 8

Populations^**a**^	Start (bp)	End (bp)	Length (bp)	Region |iHS| value^**b**^	Chromosome |iHS| value^**c**^	SNP position (bp)^**d**^	Percentage of SNP in ROH (%)	***P*** value	Nearby genes	Location (kb)^**e**^
SH	31,868	702,746	670,878	1.30	0.75	87,637	50	7.24e-05	AMY2A	D 37.10
	742,239	797,216	54,977	1.72		–	–	–	–	–
NM	198,456	643,706	445,250	1.78	0.73	553,538	54.55	6.76e-05	NTNG1	U 274.61
DZ	37,270	401,505	364,235	0.68	0.71	–	–	–	–	–
LZ	781,122	1,127,960	346,838	2.99	0.81	781,122	70	2.01e-04	NTNG1	U 47.02
						960,534	80	5.14 e-04	NTNG1	Intron
						1,019,114	80	5.57 e-04	VAV3	Intron
						1,040,079	80	5.61e-06	VAV3	Intron
						1,090,605	80	3.43 e-04	VAV3	Intron
						1,104,514	80	3.69 e-04	VAV3	Intron
						1,107,611	80	1.08 e-04	VAV3	Intron
						1,112,244	80	2.72 e-04	VAV3	Intron
LW	357,766	657,445	299,679	1.73	0.75	612,663	50	3.41e-07	NTNG1	U 215.49
						630,438	44.44	4.37e-05	NTNG1	U 197.71

^a^SH, NM, DZ, LZ and LW denote Shigatse, Nyemo, Dagze, Ningychi and Lhasa white chicken population, respectively

^b^Region |iHS| value denotes mean |iHS| value of SNPs in the studied region

^c^Chromosome |iHS| value denotes mean |iHS| value of all SNPs in the chromosome 8

^d^SNP with highest |iHS| value

^e^D and U indicates that the SNP is in the downstream and upstream of the gene, respectively

## Discussion

This study used whole genome sequences of four Tibetan chicken populations reared on the Tibetan plateau to analyze genetic diversity, run of homozygosity, genomic inbreeding and selection signatures. A composite Tibetan local breed, Lhasa white was also included in the analyses to compare results among populations. Lhasa white is a synthetic breed generated by crossing male White Leghorn and female Tibetan chickens, which has been reared for over sixty years on the Tibetan plateau.

Observed (Ho) and expected (He) heterozygosity values for SNPs with MAF ≥ 0.05 close to 0.3 were found in all populations. Similar values were reported in modern chicken populations using sequence data [15] but lower values were documented for Italian local chickens [12]. Moreover, when all SNPs were used in the analysis to avoid bias [21], we found lower Ho for all populations compared to that reported in Dutch local chickens genotyped on 60 K SNP arrays [10]. In our study, we observed slightly lower heterozygosity than expected in SH, NM, DZ and LW, suggesting subtle inbreeding in these populations. However, a little heterozygosity excess (Ho > He) was observed in the LZ population. This suggests a recent bottleneck or an isolate-breaking effect [22] which may likely be due to the recent domestication and selection process, but also due to the small sample size. Pair-wise Fst among SH, NM and DZ was less than 0.05, indicating little genetic differentiation according to Wright’s interpretation [23]. The LZ population was isolated from SH, NM and DZ populations, suggesting little or no admixture between them during its domestication. This suggestion is supported by the clear separation of LZ birds from SH, NM and DZ revealed by principal component analysis, which corroborated the earlier submission that two or more distinct Tibetan chicken populations live in the plateau [2, 4].

In our present study, the number and the distribution of ROHs identified in Tibetan native chickens were comparable to those reported in broiler chickens [14]. Furthermore, short ROHs were predominant in the Tibetan chicken populations, indicating that little deleterious inbreeding happened in Tibetan native chicken populations [24]. The relationship between the total number of ROHs and the total length of the genome covered by ROHs showed considerable variation among birds within and across populations. Similar distributions were also reported in commercial chickens [15] and other livestock species including sheep [17] and cattle [25]. Genomic data is the only reliable source to estimate the inbreeding and relatedness of populations in the absence of other data sources, such as pedigree. The proportion of the genome within ROHs (F_ROH_), genomic SNP-by-SNP inbreeding coefficient (F_GRM_), excess of homozygosity (F_HOM_) and correlation between uniting gametes (F_UNI_) were commonly accepted indicators for inbreeding assessment [26]. Herein, the ROH-based genomic inbreeding coefficients of Tibetan chicken were similar to the estimates in other Chinese local chickens studied [11], but much smaller than those in modern chickens [15, 16] and Italian local chickens [12]. Therefore, Tibetan native chickens are diverse and less affected by inbreeding, suggesting that they have maintained their natural diversity in the plateau. The correlations between F_ROH_ and F_HOM_, as well as F_UNI_ were significantly high, similar to those reported in cattle [27], pigs [28], horses [29] and modern chickens [15]. This further supports F_ROH_ as an accurate estimate of identity by descent (IBD).

Whole-genome sequencing allows detection of the ROH and its analysis enables reliable inference of the demographic history of animal populations. It also provides a new approach for the exploration and discovery of complex traits [5]. The ROH islands of Tibetan chickens and Lhasa white chickens harbored many QTLs and candidate genes controlling economically important traits, including conformation, production, egg and meat quality, digestion and absorption, reproduction and growth traits. The QTLs for the comb weight and the ileum weight harbored within the common ROH islands in the Tibetan native chicken populations might play an important role in their adaptation to high altitude. Regarding common genes located on GGA5, leucine-rich repeat-containing G protein-coupled receptor 4 (*LGR4*), enriched in the biological process of osteoblast in GO database, contributes to the regulation of energy metabolism including food intake and energy expenditure [30]. Brain-derived neurotrophic factor (*BDNF*), enriched in the positive regulation of synapse assembly is considered important for the temperature perception in chickens [31]. In rats, BDNF administration in the paraventricular nucleus reduced energy intake and decreased body weight [32]. *STAT1* and *STAT4* are members of Janus kinase (JAK)-signal transducer and activators of transcription (STAT) pathway that plays critical roles in facilitating various cellular reactions to cellular stress including hypoxia, ultraviolet light and hyperosmolarity [33]. Moreover, these genes were previously identified as ROH islands-associated genes in Mexican highland chickens [20], suggesting their critical roles in adaptation to highland. Additionally, metal ion binding was enriched in 34 of the identified genes. Although the process of how metal ion binding affects animal’s physiology and production is rarely reported, some genes enriched in the term including *VAV3*, *NOS2*, *COL3A1* and *PRKD1* were putative candidate genes associated with highland adaptation [34], implying that the metal ion binding may be associated with highland adaptation.

ROH islands might be the representative genomic regions under natural and artificial selection [35]. The iHS approach appears to be powerful for detecting ongoing selection processes for which the target allele has a moderate to high frequency within a population [36]. Our iHS analysis revealed that the common genomic region with different ROH islands on GGA5 and GGA8 overlapped with putative selection signatures in SH, NM, LZ and LW populations, indicating ongoing selective forces. Commonly identified regions by both iHS and ROH analysis harbored *AMY2A*, *NTNG1* and *VAV3* genes on GGA8. *AMY2A* encodes a member of the alpha-amylase family of proteins, which is involved in carbohydrates and glycogen metabolism, affecting growth, carcass traits and feed intake efficiency in chicken [37]. The previous report showed that *AMY2A* was under selection for metabolism, energy availability and response to thermal stress in African chickens [38]. Similar to African village conditions, chicken feeding is mainly based on scavenging, household waste and some grain supplementation in the Tibetan plateau. Therefore, carbohydrate metabolism, energy generation and transport are important traits for feeding adaptation. *NTNG1* is a responsible gene for axon and neurite growth [39]. It was also differentially expressed in a chicken hepatocellular cell line in response to stress [40]. *VAV3* is a member of the VAV gene family that plays vital role as guanosine nucleotide exchange factors for Rho GTPases and signaling adaptors downstream of protein tyrosine kinases [41]. Specifically, *VAV3* had been identified as a candidate gene associated with highland adaptation in Ethiopians [42] and Ethiopian sheep [43]. This probably resulted from its role played in the homeostasis of the cardiovascular system [44]. We therefore suggest that *VAV3* also functions putatively in the adaptation to the high altitude of Tibetan native chicken. Given that different chicken populations were reared in the Tibetan plateau for many decades, we speculated that this genomic region and the candidate genes on GGA8 might be under natural or artificial selection for adaptation to the high-altitude environment.

## Conclusions

In the present study, we used whole genome sequence data to characterize the genetic diversity and investigate the distribution of ROH across the genomes of five Tibetan indigenous chicken populations. Different LD, diversity and ROH patterns were observed in the five populations. Genetic diversity evaluated by observed heterozygosity was high in the five populations. The Nyingchi (LZ) population, which was distant from other populations had the highest proportion of long ROH fragments and ROH-based genomic inbreeding coefficient, reflecting recent inbreeding events. We identified a total of 343 ROH islands harboring 112 QTLs and 1429 genes. Five of such genes were involved in energy metabolism and STATs pathway. Specifically, ROH islands on GGA8 harbored genes including *AMY2A*, *NTNG1* and *VAV3*. This region is suggested as a candidate genomic region for adaptation to the high-altitude environment, which should be further validated in the following studies. Our findings contribute to the understanding of genetic diversity, population inbreeding and the underlying genetic mechanism of the high-altitude adaptation, and may help in the design and implementation of breeding and conservation strategies for the chickens.

## Methods

### Ethics statement

All birds were handled following the guidelines established by the Council for Animal Welfare of China. The experimental protocols were approved by the Science Research Department of the Institute of Animal Sciences, Chinese Academy of Agricultural Sciences (CAAS).

### Whole-genome sequencing and data processing

Genomic DNA was extracted from the blood of 114 female chickens. After purification and integrity verification of the DNA, whole-genome sequencing was performed using the Illumina HiSeq 2500 Sequencer (Illumina, Inc., SanDiego, CA, USA) to generate 150 bp paired-end reads. To minimize mapping errors, low-quality reads were removed using FastQC software following Yan et al. [45]. The clean reads from each bird were aligned to the chicken reference genome (Gallus gallus5.0) using the Burrows–Wheeler Alignment (BWA) tool [46] with the default parameters. Picard toolkit was subsequently used to filter out potential PCR duplicate reads. The resulting alignments were indexed using SAMtools [47] and processed according to the best practices for the Genome Analysis Toolkit (GATK) [48]. To obtain high-quality SNPs, we set a minimum quality score of 20 for both bases and mapped reads to call variants. Finally, the SNPs of each bird were combined to obtain a common set of SNP data, which was subjected to filtering with the following rigorous criteria using the GATK Variant Filtration module; (a) quality by depth > 5.0 (b) mapping quality score > 40.0 (c) FS < 60.0 (d) MQRankSum > − 12.5 (e) ReadPosRankSum > − 8.0 and (f) Filtering out any three SNPs clustered in a 10 bp window [49]. The qualified SNPs were annotated using the chicken reference genome. Finally, annotated SNP data was filtered using PLINK v1.90 software [50] with the following parameters: sample call rate > 90%, SNP call rate > 95%, and Hardy–Weinberg equilibrium *p*-value < 10^− 5^. After these quality control steps, a total of 20,385,015 SNPs distributed across 33 autosomes in 105 birds were obtained for subsequent analyses. Among these birds, 20, 32, 25, 10 and 18 belonged to Shigatse, Nyemo, Dagze, Nyingchi and Lhasa white population, respectively.

### Genetic diversity analysis

The filtered SNPs were further pruned to obtain independent SNP markers using the ‘-indep-pairwise’ option, with a window size of 50 SNPs, a step of 10 SNPs, and r^2^ threshold of 0.2. Principal components (PC) analysis was carried out with the pruned data in PLINK, and the first 2 PCs were extracted and plotted using R software. The observed heterozygosity (Ho) and the expected heterozygosity (He) were estimated based on allele frequency of all eligible SNPs, SNPs with minor allele frequency (MAF) ≥0.01 and SNPs with MAF ≥ 0.05, respectively, using ‘-hardy’ option in PLINK. Multiple-locus heterozygosity (MLH) was also calculated for each bird with formula $\frac{N-\mathrm{O}}{\mathrm{N}}$ [51], where *O* is the number of observed homozygotes, and *N* is the number of non-missing autosomal SNPs. The *O* and *N* were calculated using ‘-het’ option in PLINK. Further, PopLDdecay [52] was used to estimate linkage disequilibrium (LD) using SNPs with MAF ≥0.01, and the LD decay was plotted in R software. Moreover, fixation index (Fst) value of SNPs across the genomes were estimated using VCFtools [53], and then averaged to evaluate the relatedness among populations.

### Runs of Homozygosity (ROH) detection

Prior to the ROH detection, the eligible SNPs with MAF ≥0.01 were separately filtered for each population. Long homozygous fragments were scanned in the pruned data using PLINK according to the following parameters: the minimum number of 50 SNPs in a ROH (−homozyg-snp 50), sliding windows of 50 SNPs (−homozyg-window-snp 50), allowance for not more than 5 missing SNPs (−homozyg-window-missing 5) and three heterozygous SNPs per window (−homozyg-window-het 3). The minimum length of an ROH segment was 300 kb (−homozyg-kb 300). The minimum SNP density was 1 SNP per 50 kb (−homozyg-density 50), and the maximum gap between two consecutive SNPs was 1000 kb (−homozyg-gap 1000). Finally, the rate in which a SNP was included in the total of sliding windows was at least 0.05 (−homozyg-window-threshold 0.05). After the run, the identified ROHs were classified according to their size into small (< 1 Mb), medium (1 ~ 3 Mb) and large (> 3 Mb) as previously delineated in chickens [10].

### Genomic inbreeding analysis

Genomic inbreeding based on ROH (F_ROH_) was estimated using PLINK according to previous methods [54]. The F_ROH_ for each bird was calculated as $\frac{\sum_i{L}_{ROH_i}}{L_{aut}}$, where ${L}_{ROH_i}$ is the length of ROH of *i* th individual, and *L*_*aut*_ is the genome length of autosome covered by the SNPs in the sequence data. The inbreeding coefficient for an individual based on homozygous SNPs (F_HOM_) was computed as $\frac{\left(O-E\right)}{\left(L-E\right)}$, where *O* is the number of observed homozygotes, *E* is the number of expected homozygotes by chance, and *L* is the number of non-missing autosomal SNPs. Genomic SNP-by-SNP inbreeding coefficient (F_GRM_) and the correlation between uniting gametes (F_UNI_) were computed in GCTA software as previously reported [55]. Pair-wise correlations between these inbreeding coefficients were estimated by the Pearson method.

### Identification of common ROH and gene annotation

To identify the genomic regions that harbored common ROHs across the five chicken populations, we estimated the occurrences of SNPs in ROHs by counting the number of times when the SNP was detected in those ROHs using detectRUN package [56] implemented in R. The genomic regions commonly associated with ROHs were screened by selecting the top 1% SNPs observed in ROHs. Adjacent SNPs that met this threshold were merged into genomic regions named ROH islands. Based on these consensus regions, we annotated QTL based on the chicken QTLdb using ‘-wa’ and ‘-wb’ options in BEDTools [57]. The ROH islands were also annotated with Gallus gallus5.0 genome assembly using the Ensembl BioMart [58] by extracting intersected and overlapped genes. Functional annotations and enrichments of the identified genes within the ROH islands were further carried out in the DAVID platform, and the chicken annotation file was set as background to identify significant (*P* < 0.05) GO terms and KEGG pathways.

### Selection signatures analysis

To detect selection signatures in each ROH island, the integrated haplotype score (iHS) was calculated within each population. The iHS is a measure of the amount of extended haplotype homozygosity at a given SNP, that uses phased genotypes to identify putative regions of recent or ongoing positive selection in genomes [59]. Herein, the haplotype was phased using SHAPEIT [60] with recombination rate 0.01 as previously used for chicken genome [61]. The derived haplotypes were then analyzed using the rehh (v2.0) R package [62]. The iHS score was computed for each SNP and further standardized to a *P* value with the following formula *p*_*iHS*_ = −*log*_10_(1 − 2 | ∅(*iHS*) − 0.5| ), where ∅(*iHS*) represents the Gaussian cumulative distribution function, and *p*_*iHS*_ is the two sided *P* value associated with the neutral hypothesis of no selection [62]. The *p*_*iHS*_ higher than a threshold of 0.1% were considered as putative signatures of selection. Due to the limit of computing time and lack of accurate recombination rate of studied chickens, we only ran the selection signature analysis for candidate regions on GGA5 and GGA8 in the present study.

## Supplementary Information

**Additional file 1.**

## Data Availability

The data and computing programs used in this manuscript are available from the corresponding authors on request.

## Abbreviations

SH: Shigatse
NM: Nyemo
DZ: Dagze
LZ: Nyingchi
LW: Lhasa white
ROH: Run of homozygosity
F_ROH_: ROH-based inbreeding coefficient
LD: Linkage disequilibrium
BWA: Burrows–Wheeler Alignment
GATK: Genome Analysis Toolkit
Ho: Observed heterozygosity
He: Expected heterozygosity
F_GRM_: Genomic SNP-by-SNP inbreeding coefficient
F_HOM_: Excess of homozygosity
F_UNI_: Correlation between uniting gametes
MAF: Minor allele frequency
iHS: Integrated haplotype score
GO: Gene ontology
*LGR4*: Leucine-rich repeat-containing G protein-coupled receptor 4
*BDNF*: Brain-derived neurotrophic factor
*AMY2A*: Amylase Alpha 2A
NTNG1: Netrin G1
*VAV3*: Vav Guanine Nucleotide Exchange Factor 3
