# Supplementary material for: Genome-wide run of homozygosity analysis reveals candidate genomic regions associated with environmental adaptations of Tibetan native chickens
Source: BMC Genomics. 2022 Jan 31;23:91. doi: 10.1186/s12864-021-08280-z (PMC8805376; doi:10.1186/s12864-021-08280-z)
Supplement: Supplementary file 1 — Additional file 1. [file 12864_2021_8280_MOESM1_ESM.zip › Supplementary File Legends.docx]

**Genome-wide run of homozygosity analysis reveals candidate genomic regions associated with environmental adaptations of Tibetan native chickens**

**Jingwei Yuan^1^, Shijun Li^2^, Zheya Sheng^2^, Meikun Zhang^3^, Xuming Liu^3^, Zhengdong Yuan^3^, Ning Yang^4^, Jilan Chen^1*^**

^1^Institute of Animal Sciences, Chinese Academy of Agricultural Sciences, Beijing 100193, China.

^2^Key Laboratory of Agricultural Animal Genetics, Breeding and Reproduction of Ministry of Education, Huazhong Agricultural University, Wuhan 430070, China

^3^DQY Ecological Co. Ltd., Beijing 100094, China

^4^National Engineering Laboratory for Animal Breeding, College of Animal Science and Technology, China Agricultural University, Beijing 100193, China

^*^Corresponding author: chen.jilan@163.com

Email address:

Jingwei Yuan: yuanjingwei@caas.cn

Shijun Li: lishijun@mail.hzau.edu.cn

Zheya Sheng: zheya.sheng@mail.hzau.edu.cn

Meikun Zhang: zmk5678@126.com

Xuming Liu: liuxuming@dqy.com.cn

Zhengdong Yuan: yuanzhengdong@dqy.com.cn

Ning Yang: nyang@cau.edu.cn

Jilan Chen: chen.jilan@163.com

**Supplementary files**

**Figure S1. Linkage disequilibrium decay (a) and Principal component analysis (b) plot of the genetic diversity for 5 Tibetan native chicken populations.**

**
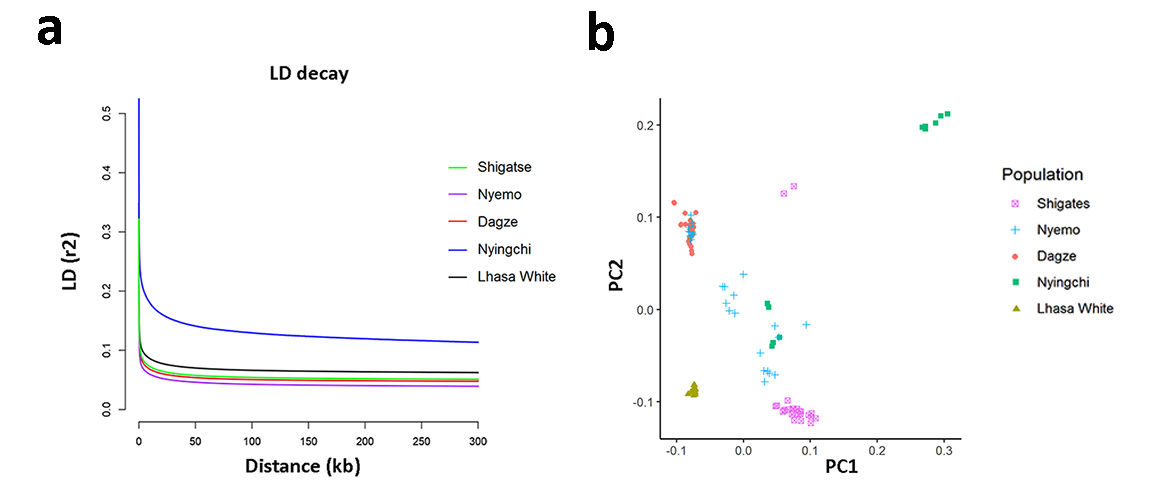
**

**Figure S2. Chromosome-wide distribution of selection signatures detected by iHS on Chromosome 5 for 5 Tibetan native chicken populations.**


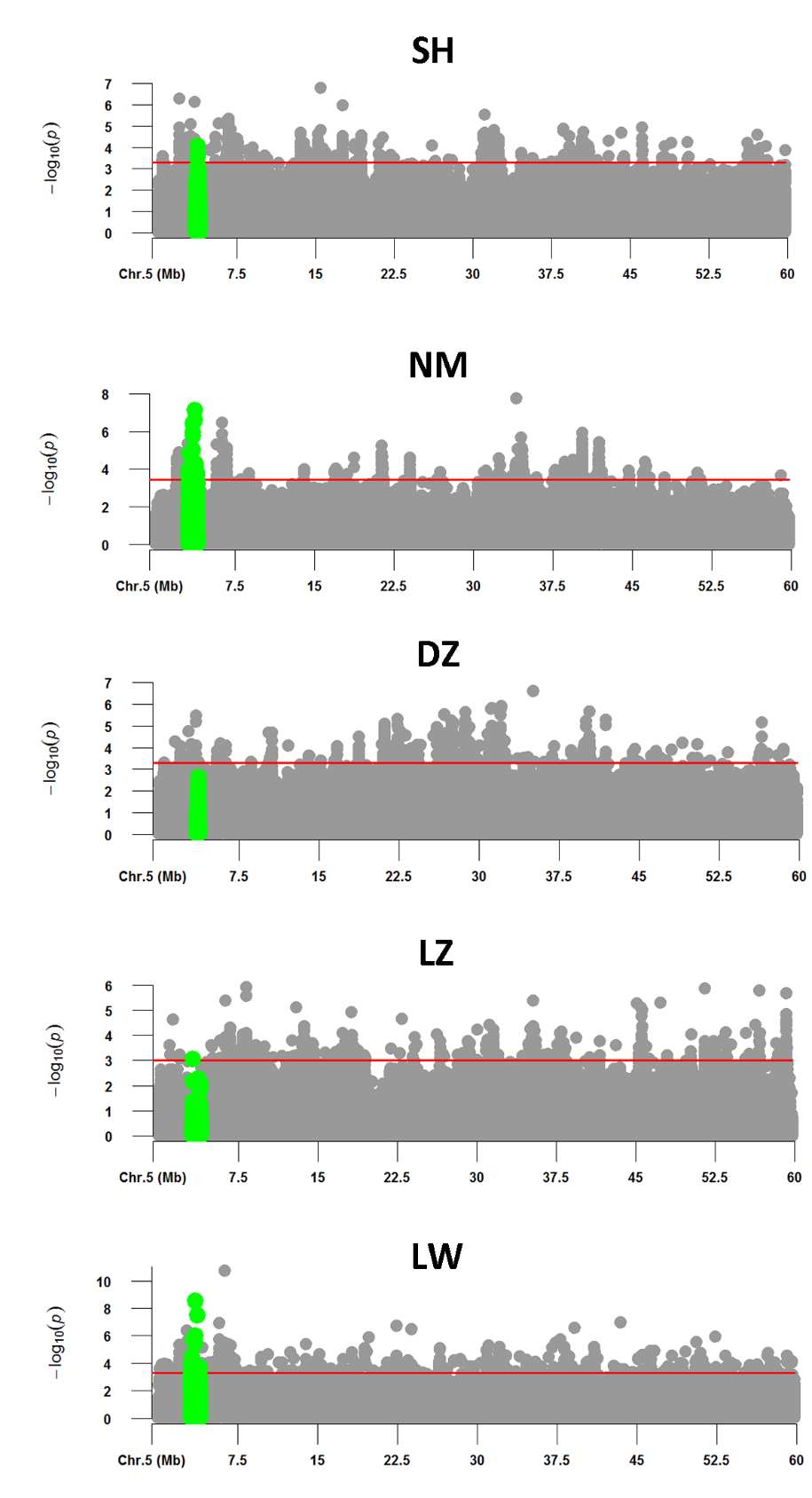


The red line represents the threshold levels of SNPs with iHS value ranked top 0.1%. The green dots represented SNPs located in the studied region and other dots were showed in grey. SH, NM, DZ, LZ and LW denote Shigatse, Nyemo, Dagze, Ningychi and Lhasa white chicken population, respectively.

**Table S1. Pairwise Fst between chicken populations.** SH, NM, DZ, LZ and LW denote Shigatse, Nyemo, Dagze, Ningychi and Lhasa white chicken population, respectively.

**Table S2. Gene ontology (GO) term for genes harboured in ROH island**

**Table S3. ROH island overlapped with reported QTLs for 5 populations.** SH, NM, DZ, LZ and LW denote Shigatse, Nyemo, Dagze, Ningychi and Lhasa white chicken population, respectively.
